# Intellectual Development in Mexican Preterm Children at Risk of Perinatal Brain Damage: A Longitudinal Study

**DOI:** 10.3390/children11060652

**Published:** 2024-05-28

**Authors:** Cynthia Torres-González, Josefina Ricardo-Garcell, Daniel Alvarez-Núñez, Gilberto Galindo-Aldana

**Affiliations:** 1 Faculty of Administrative, Social, and Engineering Sciences, Universidad Autónoma de Baja California, State Hwy No. 3, Guadalupe Victoria, Mexicali 21720, Baja California, Mexico; gilberto.galindo.aldana@uabc.edu.mx; 2 Neurodevelopmental Research Unit “Augusto Fernandez Guardiola”, Institute of Neurobiology, Autonomous University of Mexico, Boulevard Juriquilla 3001, La Mesa, Juriquilla 76230, Querétaro, Mexico; 3 CETyS University, Calzada CETYS s/n. Col. Rivera, Mexicali 21259, Baja California, Mexico; daniel.alvarez@cetys.mx

**Keywords:** intellectual development, preterm birth, working memory, perinatal risk factor for brain damage

## Abstract

Preterm birth accounts for about 10% of births worldwide. Studying risk factors for perinatal brain damage is essential, as findings suggest that almost 20% of disabilities are linked to risks in the early stages of development. This research aimed to study longitudinal changes in intelligence from 6 to 8 years of age in a sample of 39 preterm children with a history of risk of brain damage and a control group of 35 children born at term. The Wechsler Intelligence Scale (WISC-IV) was used to measure cognitive ability at six, seven, and eight years old. The results showed that the preterm group obtained significantly lower scores than the control group. The working memory indicator significantly affected the interaction between age and prematurity. We consider it crucial to expand the knowledge we have about the neurocognitive development of premature infants, both in specific cognitive domains and in age ranges, so that the information obtained can help predict the probability of presenting cognitive alterations from early stages. This, therefore, helps in implementing intervention strategies and programs based on scientific evidence, and their design is complemented by clinical experience and empirical and theoretical knowledge of the different professionals involved in infant cognitive intervention.

## 1. Introduction

According to the World Health Organization (WHO), preterm birth occurs before 37 weeks gestation (before 259 days from the date of the last menstrual period) [1]. It is a condition defined by the failure to reach the expected gestational time and not by the presence of specific symptoms or signs [2]. Thus, preterm birth can be seen as a result of complications during pregnancy that anticipate the time of birth or as a medical alternative to avoid a greater risk to the fetus or the mother [2].

Gestational age is the most frequent criterion for the classification of prematurity, grouped into: late when it occurs between 36 and 32 weeks of gestation; moderate, between 31 and 29 weeks; and extreme when it occurs before 28 weeks. Although the risk of survival and morbidity increases the lower the gestational age is, chances are present in all categories [1]. Its etiology is heterogeneous, characterized by a complex interaction between factors that include five components: (1) maternal conditions before or during labor, (2) fetal conditions, (3) pathological placental conditions, (4) signs of the onset of labor, and (5) type of delivery [3,4]. According to data from international organizations such as the WHO and the United Nations, about 30% of cases occur due to early labor or cesarean section for medical or personal reasons. Meanwhile, approximately 70% occur spontaneously. Among the most frequent causes are multiple pregnancies, infectious or chronic maternal health conditions, and genetic factors [5,6]. However, in many cases, the reason is not determined [1,2].

Prematurity is a frequent phenomenon and represents the leading cause of perinatal mortality [7]. Different studies have reported figures suggesting that between 10% and 16% of births occurring annually in the world are premature, representing about 15 million cases, with most of these occurring in regions such as South Asia and sub-Saharan Africa [8,9,10].

The statistics in Mexico are similar to those reported worldwide, with 7.04% of total births registered in 2014 [11]. However, it is essential to note that these data correspond to births registered in health institutions, so figures on births occurring outside health facilities are unknown. Studying the risk factors for pre- and perinatal brain damage, including prematurity, is of interest to neuroscience and neuropsychology since they occur during a critical period for brain maturation and, therefore, cognitive, socioemotional, and physical development [12]. Furthermore, the findings suggest that some disabilities and cognitive impairments originate in the early stages of life due to biological, psychosocial, and environmental risks [13]. These conditions are exacerbated in developing countries since it is estimated that about 43% of children under five years old do not reach their potential, increasing the likelihood of presenting mental health problems [14,15].

In the particular case of children with a history of prematurity, it is recognized that although mortality rates have decreased in recent decades, the morbidity associated with long-term neurocognitive problems has remained [16]. The hypomyelination, neuronal, and axonal damage that characterize the preterm brain has a high potential to cause high prevalence but low severity cognitive impairments [17]. Studies suggest that these difficulties begin to be more noticeable from school age onwards and are wide-ranging, including motor problems, alterations in visuospatial processing, memory, language, executive functioning, and intelligence [18,19,20,21,22,23].

Statistics suggest that about 70% of children with these backgrounds require exceptional education support, representing a significant economic and social burden on families and communities [24]. Developmental monitoring of preterm infants during infancy usually includes measurements of general intellectual functioning. Intellectual quotient (IQ) measures are generally the basis for cognitive assessment, which can guide the search for possible deficits in more specific functions. A linear relationship between weeks of gestation and IQ has been reported, and the rate of severe intellectual disability in this population is estimated to be around 10–50% of cases [3,22,25,26,27].

The cognitive alterations described in preterm infants as a consequence of medical and neurological complications have repercussions on the quality of life of individuals in the medium and long term. They have been associated with the presence of symptoms of neurodevelopmental disorders, such as attention deficit hyperactivity disorder, autism spectrum disorder, learning problems, poor academic performance, and risk behaviors in adolescence such as substance abuse, involvement in criminal activities, early pregnancies, school dropout, etc. [18,28,29,30,31].

Most of these studies use cross-sectional designs to examine different stages of development in different participants, so conclusions about development are based on indirect inferences. Longitudinal studies in preterm samples are scarce due to the inherent difficulties of this research design; however, one of their advantages is that they allow for the detection and measurement of the evolutionary change in cognitive development.

This study aimed to longitudinally study the impact of prematurity and risk factors for perinatal brain damage on intelligence development in a sample of children at six, seven, and eight years of age with and without a history of prematurity.

## 2. Materials and Methods

The data presented in this research article belong to a prospective longitudinal study of a group of preterm children with risk factors for perinatal brain damage and a group of children born at term without risk factors, who were evaluated annually at six, seven, and eight years old.

### 2.1. Participants

A total of 74 six-year-old children were recruited at the beginning of the study; 39 of them formed the preterm group born between 28 and 36 weeks of gestation (31.7 ± 0.8), and the remaining 35 formed the control group born between 38 and 40 weeks of pregnancy (38.7 ± 0.85). The participants in the preterm group were part of the general research protocol of the Neurodevelopment Research Unity “Augusto Fernández Guardiola” (UIND by its Spanish acronym) of the Neurobiology Institute (NBI), in the Autonomous University of México, and were selected under the following criteria: birth between 28 and 36 weeks of gestation, obtaining a score classified in the categories of normal development or mild–moderate delay in the cognitive and motor scales of the Bayley Development Scale (BSID-II) at one year of age, without severe motor or sensory disability, and having a structural MRI in the first year of age without evidence of clinically significant brain lesions.

The control group participants were recruited through public and private preschool and elementary schools, as well as direct invitation from the NBI staff. The criteria for the selection of participants in this group were: birth between 37 and 41 weeks of gestation, no history of risk factors for perinatal brain damage, no known psychiatric or neurological diagnosis, attendance at a regular school with an average level of performance, no severe motor or sensory deficits, and no behavioral problems. Table 1 shows the demographic characteristics of the participants. Each participant’s parents properly signed informed consent, agreed to voluntarily participate in the study, and were informed about their right to leave the investigation at any moment.

### 2.2. Instruments and Procedure

Intelligence assessment: this function was evaluated using the Wechsler Intelligence Scale for Children, fourth edition (WISC-IV), in its version with norms for Mexico [32]. This test consists of 15 subscales that are organized into four indexes: the Verbal Comprehension Index (VCI), the Perceptual Reasoning Index (PRI), the Working Memory Index (WMI), and the Processing Speed Index (PSI), as well as a total intelligence quotient (IQ). The test applies to children in a rank between the ages of 6 and 16 years 11 months. The ten mandatory scales were used in standard form to obtain the indexes (similarities, vocabulary, comprehension, cube design, concepts with drawings, matrices, digit repetition, number and letter ordering, clues, and symbol search). The neuropsychologist completed the study application, and two psychology students were about to graduate. The reliability coefficients for the subscales ranged from 0.70 to 0.89, while the composite scores ranged from 0.88 to 0.97.

The sample for the premature infant group was selected from an exhaustive review of the database of children who entered the UIND protocol; 102 children were pre-selected who met the previously mentioned inclusion criteria, and the parents were contacted by telephone to invite them to participate; 50 of them agreed to participate. The evaluation sessions were conducted individually in a psychological office within the UIND facilities. Six of the fifty children initially evaluated were excluded because they did not meet the inclusion criteria, so the sample in the first evaluation period comprised 44 participants. In the 7-year evaluation, it was possible to contact only 40 families, and the procedure was the same as in the initial evaluation.

Finally, for the 8-year evaluation, 39 children participated, and the same procedure was followed to apply the instruments. In the three evaluation periods, a written report of the results was given to the participant’s parents. To recruit the control group, three regular schools in Querétaro were contacted and asked to pre-select among the students in the third grade of preschool and first grade of elementary school with regular academic performance. Then, the children’s parents were invited to participate in the study. Thus, the total number of participants in the initial period (six years) who met the requirements and whose parents signed the informed consent was 49. The evaluation was carried out in designated cubicles within the facilities of the participating schools and the UIND in individual sessions. In each period evaluated, a written report of the results was given to the participant’s parents.

### 2.3. Data Analysis

Data analysis was performed using the SPSS.25 statistical program (IBM). An exploration of the data was carried out, and the cases whose values were identified as extreme in the box plots were eliminated; the sample distribution was standard in each of the cognitive variables evaluated according to the results of the Shapiro–Wilk test (*p* > 0.05). A repeated measure mixed ANOVA (2X3) was performed to determine the existence of significant differences in the scores obtained in the indices measured by the test: verbal comprehension, perceptual reasoning, working memory, processing speed, and total IQ between the groups (preterm and full-term) and throughout the three evaluation periods (6, 7, and 8 years). Univariate and multivariate normality assumptions were tested. The equality of the covariance matrices and the absence of multicollinearity and post hoc analysis with Bonferroni adjustment were performed for each measured index.

### 2.4. Ethical Considerations

The information from participants was anonymously driven and only known by the principal investigator of this research; the participants’ parents were informed about the objectives of the study and were free to participate or decline the procedures voluntarily. The protocol was approved by the Autonomous University of Baja California, Faculty of Administrative, Social and Engineering Sciences Institutional Review Board.

## 3. Results

The results obtained by each of the groups in the WISC-IV in the three evaluation phases are shown in Table 2.

### 3.1. Verbal Comprehension

**Figure 1:**
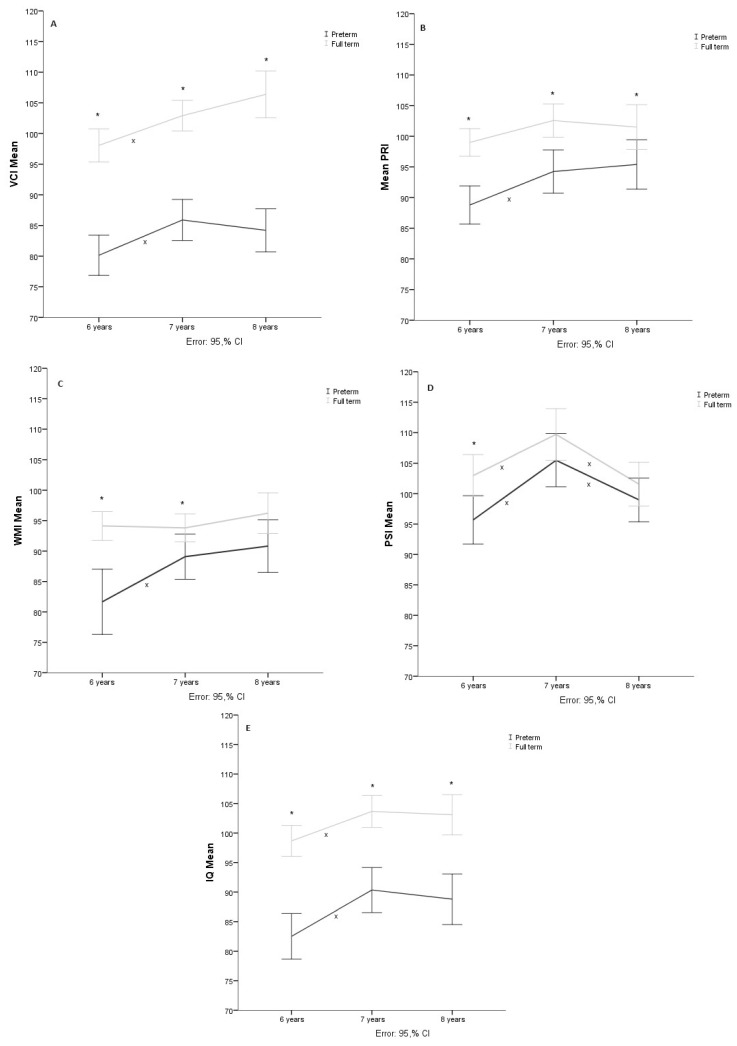
Note. (**A**): Verbal Comprehension Index (VCI), (**B**): Perceptive Reasoning Index (PRI), (**C**): Working Memory Index (WMI), (**D**): Processing Speed Index (PSI), (**E**): Full Scale Intellectual Quotient (IQ). * Shows intergroup significative differences *p* < 0.05; x shows intragroup significative differences *p* < 0.05 in the post hoc analysis.

Three cases with extreme values were eliminated—two of them correspond to the control group, whose scores at seven years were very high compared to the rest of the group, and one participant from the preterm group, whose score at six years was very low—so the sample comprised 71 participants (38 preterm and 33 terms). The Shapiro–Wilk test showed a normal distribution (*p* > 0.05). Therefore, the sphericity of the data measured through Mauchly’s test of sphericity (*X*^2^ = 0.353, *p* = 0.838) could be assumed. The repeated measures mixed ANOVA showed that the age at which the evaluation was performed produced significant changes in the verbal comprehension index score (F(138, 6190.5) = 17.712, *p* < 0.05, $\eta^{2}$ = 0.204). However, the group*age interaction analysis did not show significant effects (F(2, 268.7) = 2.996, *p* = 0.053, $\eta^{2}$ = 0.204). Post hoc analysis by Bonferroni adjustment showed significant differences (*p* < 0.05) between the groups in the three evaluation phases, as well as intragroup differences (*p* < 0.05) between 6 and 7 years in both groups (see Figure 1A).

### 3.2. Perceptual Reasoning

Data from 67 of the participants (37 preterms and 30 terms) were analyzed since seven cases were eliminated for presenting extreme values; the Shapiro–Wilk test showed a normal distribution (*p* > 0.05). However, the sphericity of the data measured by Mauchly’s test *X*^2^ = 11.322, *p* = 0.003 could not be assumed, so the correction of the degrees of freedom with the Huynh–Feldt sphericity estimate (ϵ = 0.895) was considered. The repeated measures mixed ANOVA showed that age at evaluation significantly changed the IRP score (F(116.38, 5091.88) = 11.609, *p* < 0.001, $\eta^{2}$ = 0.152). At the same time, the group*age interaction analysis showed no significant changes (F(1.791, 141.03) = 1.8, *p* = 0.175, $\eta^{2}$ = 0.027). On the other hand, post hoc analysis showed significant differences (*p* < 0.05) between the groups in the three evaluation phases, as well as significant differences (*p* < 0.05) within groups between 6 and 7 years for the preterm group but not for the term group (see Figure 1B).

### 3.3. Working Memory

The results of 69 participants (38 preterms and 31 terms) were analyzed, and the Shapiro–Wilk test showed a normal distribution (*p* > 0.05). Sphericity of the data was assumed measured through Mauchly’s test of sphericity (*X*^2^ = 5.620, *p* = 0.060). The repeated measures mixed ANOVA showed that age at evaluation produced significant changes (F(134, 6655.57)=11.13, *p* < 0.001, $\eta^{2}$ = 0.142). In addition, the group*age interaction showed a significant effect (F(2, 627.63) = 6.31, = 0.002, $\eta^{2}$ = 0.086). On the other hand, the results of the post hoc analysis showed significant differences between the groups in the first two evaluation stages. The preterm group showed significantly different scores (*p* < 0.05) between 6 and 7 years of age but not from 7 to 8 years of age, while the group of children born at term did not show significant differences (*p* > 0.05) between ages (See Figure 1C).

### 3.4. Processing Speed

The analysis was performed with 69 participants (35 preterms and 34 terms) after five cases were eliminated for presenting extreme values. The Shapiro–Wilk test showed that there was a normal distribution (*p* < 0.05). The results of the Mauchly test showed that the sphericity was fulfilled (*X*^2^ = 2.854, *p* = 0.240). Repeated measures mixed ANOVA showed that age at evaluation produced significant changes (F(134, 9716.20)= 19.499, *p* < 0.001, $\eta^{2}$ = 0.225), but the group*age interaction yielded no evidence of substantial changes (F(2, 196.176) = 1.353, *p* = 0.262, $\eta^{2}$ = 0.020). Post hoc analyses revealed significant differences (*p* < 0.05) between groups at six years. In addition, both the preterm and term group obtained significantly different scores (*p* < 0.05) between the three ages (see Figure 1D).

### 3.5. Total Intelligence Quotient

Table 3 shows the Total Intellectual Quotient repeated measures analysis results of 71 participants (38 preterms and 33 terms). The results of the Shapiro–Wilk test showed a normal distribution (*p* > 0.05). The sphericity of the data could not be assumed according to Mauchly’s test (*X*^2^ = 6.999 *p* = 0.030), so degrees of freedom were corrected with Huynh–Feldt’s estimate of sphericity (ϵ = 0.948). The repeated measures mixed ANOVA showed that there is an effect of age (F(130.8, 4506.198) = 25.511, *p* < 0.001, $\eta^{2}$ = 0.270) but not of the age*group interaction (F(1.896, 72.205) = 1.106, *p* = 0.332, $\eta^{2}$ = 0.016). Post hoc analysis evidenced significant (*p* < 0.05) intragroup changes in total IQ in the two groups between the ages of six and seven. At the same time, intergroup differences were significantly (*p* < 0.05) present in the three ages evaluated (see Figure 1E).

## 4. Discussion

The objective of this research was to longitudinally study the impact of prematurity and risk factors for perinatal brain damage on intelligence development. A preterm and term children sample was evaluated using the WISC-IV at 6, 7, and 8 years. A comparison of the demographic data showed that the groups were different in the expected variables (weeks of gestation, birth weight, and risk factors). No differences were observed in variables such as the ages at which they were evaluated in each period or maternal schooling, which guarantees that the differences between the groups are not due to more significant maturation due to age or disparities in socioeconomic level when choosing the sample.

Among the indicators considered in the evaluation of this study, the working memory index showed a significant effect of the interaction between age and group. The data showed significant changes in the scores obtained in this index throughout the three ages in the preterm participants but not in the control group. In addition, directionality analyses showed that in all three assessments, the control group’s scores were significantly higher than those of the preterm infants.

Previous studies have reported such differences in preterm and non-preterm children. An example is the study conducted by Fitzpatrick and collaborators [33], who reported significant differences in the performance of a spatial working memory task between moderate preterm and full-term children at 11 years of age. Similar findings are reported by Korpela et al. [34], who compared the performance of 95 preterm children at 11 years of age on working memory tasks and the WISC-IV; in their study, the group was divided into three subgroups according to the degree of brain pathology (identified on neonatal MRI): normal, minor, or severe. In addition to comparisons between the three groups, performance was compared against the norms of the different tests. This study showed that preterm infants (regardless of a subgroup) with an average level of cognitive performance on the intelligence test performed significantly worse than test norms, particularly on tasks related to the central executive and visuospatial agenda. Meanwhile, the group with the most severe neonatal brain lesions had the worst results.

Ford et al. [35] evaluated 35 preterm children aged 7 to 11 years and 37 term-born children of the same ages. The tests used the DSM-IV regression digit repetition task (the same task we used in our study). According to the results of the statistical analyses, there were significant differences between the groups in this domain, suggesting differences in prospective memory.

More recent similar results were reported by Kaul et al. [36], who contrasted the WICS-IV performance of 359 extremely preterm children with the performance of 367 non-preterm children. The mean age of the participants was 6.5 years, and the results showed that the most significant differences between the groups were perceptual reasoning and working memory.

The indices of verbal comprehension, perceptual reasoning, processing speed, and total IQ only showed significant effects of age. Differences between the groups were present at all three ages in the verbal comprehension index, perceptual reasoning, and total IQ, while working memory and processing speed showed differences only at 6 and 7 years and six years, respectively. These data are consistent with studies noting that preterm infants tend to have significantly lower scores than full-term infants on intelligence tests [36,37,38,39,40]; furthermore, studies report that these differences are maintained over time, holding even into adulthood [41,42]. A domain that may be particularly interesting is processing speed since the main neurological alterations reported in preterm infants are associated with lesions in the white matter [43,44]. In neurodevelopmental pathology, a spectrum of white matter injuries is acknowledged, each distinguished by unique neuropathological features comprising a blend of deleterious and developmental anomalies [45]. This brain immaturity affects oligodendrocyte precursor cells, representing the cells responsible for brain myelination [45,46]. The presence of these characteristics potentially gives rise to disruptions in the brain’s structural connectivity [47,48].

In our study, no interactions between age and group were found, and in both groups, a pattern of decreasing tendency was found between the ages of 7 and 8. These changes described in processing speed contradict the findings of other studies indicating that processing speed increases substantially during middle childhood and less markedly in late childhood. In both groups, scores on this index tended to decline. However, given that these changes remain within the parameters of normality in both groups, it is possible to affirm that this is not a pathological phenomenon, but a change in the dynamic interaction between cognitive processes. Thus, for example, it has been described that processing speed may decrease in the face of changes in the executive control demands of the task [49,50].

In general, the results of our study, related to intellectual activity, suggest that intelligence and its different components change throughout childhood, regardless of the condition at birth. However, it is essential to note that although the trajectories found have similar characteristics between the groups, in the case of the term infants, the mean scores for each of the indicators evaluated always remained within a performance classification within the normal parameters for their age (range of 94.4 to 110) according to the test, while in the preterm group, the mean scores were mainly in the low average (range from 80.9 to 101.8). The evaluation results of the preterm group participants suggest a slight decrease in intellectual capacity compared to the control group. The developmental trajectory drawn in the three age cutoffs evaluated is closer to a pattern of delay than to one of impairment since, in almost all the components evaluated, the differences in the scores reached at eight years of age are smaller than the differences between the groups at six years of age. These data are consistent with research work suggesting that preterm intelligence is within the parameters of normal but significantly below-term births [3,22,51,52].

Roze et al. [53] suggest that other cognitive deficits often accompany low IQ scores obtained by preterm infants, making it relevant to perform comprehensive neuropsychological assessments systematically throughout development. These characteristics of cognitive functioning are associated with structural changes and brain connectivity. Low intelligence test scores correlate with decreased connectivity between the left lateral occipital region and parietal lobes [54].

Finally, we consider that it is crucial to expand the knowledge we have about the neurocognitive development of premature infants, both in specific cognitive domains and in age ranges, so that the information obtained helps to predict the probability of presenting cognitive alterations from early stages.

This would allow for the implementation of intervention strategies and programs based on scientific evidence, such as neurohabilitation programs based on the Katona method [55,56] and neuropsychological programs focused on specific cognitive processes [57] that are complemented in their design by the clinical experience, empirical, and theoretical knowledge of the different professionals involved in child cognitive intervention.

We also find it relevant to consider the interaction of the changes found with other psychosocial variables, such as maternal schooling, which may have a modulating effect, as has been reported in works such as that of Wang et al. [23].

## 5. Limitations and Future Considerations

The generalization of the data may be limited by two conditions of this study. This includes the sample size in both groups and the fact that the participants of the preterm group belong to a broader research protocol that involves the follow-up of their neurodevelopment and the implementation of neurohabilitation techniques for their intervention from two months of age, a condition that is not common to the preterm population in Mexico. The results regarding the alterations presented throughout the period studied may be attenuated by that factor. Nevertheless, these limitations open the door for future research in which neuropsychological data are correlated with data obtained by means of techniques such as electrophysiology, detecting non-optimal brains’ bioelectrical oscillations according to specific ages, and their relation with cognitive domains, such as motor, language, communication, or social skills. For example, alpha rhythm development and its relation with self-regulation and control, beta–theta proportion rates and their relation with emotional regulation, as well as further comprehension of brains’ bioelectrical coherence between cortical and subcortical regions. In addition, other possible confounding variables should be considered in longitudinal studies for further control of their effects on children’s brain development, according to the approach of the present study; for instance, socioeconomic and environmental conditions, learning environment, and genetic variables are suggested.

## Figures and Tables

**Table 1:** Participants’ demographics.

	Preterm (*n* = 39)		Full Term (*n* = 35)		*X* ^2^
**Variable**	**n**	**%**	**n**	**%**	
Sex					0.525
Girls	20	51.3	15	42.9	
Boys	19	48.7	20	57.1	
Dominance					1.79
Right-handed	33	84.6	33	94.3	
Left-handed	2.29	0.970	2.62	0.651	
Schooling at the start of the study					12.7 *
3rd Preschool	25	64	23	8	
1st Elementary	14	36	77	27	
	**Mean**	**SD**	**Mean**	**SD**	**Mann–Whitney’s** ***U***
Gestational age	31.7	2.8	38.7	0.85	12.0 *
Birth weight (grams)	1645.77	463	3204.4	553	0.0 *
Maternal education (yrs)	15.1	4.1	15.3	4	675.5
Risk factors (frequency)	6	3	-	-	-
Age of assessment (months)					
1st Assessment	74.7	4.3	76.3	5.5	546.5
2nd Assessment	87	3.7	87.8	4.4	595.5
3rd Assessment	100.1	3.4	100.1	3.5	671.5

Note: Superior panel shows *X*^2^ comparisons for nominal and ordinal demographic variables. The inferior panel shows Mann–Whitney’s *U* results for continuous demographic variables. * *p* < 0.05.

**Table 2:** Composite scores in the Wechsler Intelligence Scale for Children (WISC-IV).

			Preterm (*n* = 39)			Full Term (*n* = 35)		
**Index**	**Age**	**Mean**	**SD**	**CI %95**	**Mean**	**SD**	**CI %95**	*****p*****
VCI								
	6 years	80.9	10.9	77.3–84.4	98.1	7.4	95.5–100.6	0.000 *
	7 years	86.2	10.2	82.9–89.5	103.2	7.9	100.5–105.9	0.000 *
	8 years	84.5	10.7	81–88	105.7	11.4	101.5–109.4	0.000 *
PRI								
	6 years	87.3	11.1	83.6–90.9	99.3	7.8	96.6–102	0.000 *
	7 years	93.0	11.7	89.1–96.8	104.3	9.5	101.1–107.6	0.001 *
	8 years	93.6	13.9	89.1–98.2	103.8	11.5	99.9–107.8	0.029 *
WMI								
	6 years	81.3	16.2	76–86.5	94.4	6.3	92.2–96.6	0.000 *
	7 years	88.1	12.5	84.1– 92.2	96.2	9.0	93.1–99.3	0.041 *
	8 years	89.8	14.2	85.2–94.4	97.2	9.0	94.1–100.3	0.056
PSI								
	6 years	93.3	13.4	88.9–97.6	103.9	11.1	100–107.7	0.006
	7 years	101.8	16.5	96.4–107.2	110	12.1	105.9–102.7	0.163
	8 years	95.2	15.0	90.3–100.1	101.5	10.1	98–105	0.305
IQ								
	6 years	81.8	12.4	77.8–85.8	98.77	7.1	96.3–101.2	0.000 *
	7 years	89.4	12.5	85.4–93.5	104.9	9.0	101.8–108.0	0.002 *
	8 years	87.9	13.8	83.4–92.4	103.6	9.5	100.3–106.9	0.001 *

Note: VCI = Verbal Comprehension Index, PRI = Perceptual Reasoning Index, WMI = Working Memory Index, PSI = Processing speed Index, IQ = Full Scale Intellectual Quotient. The *p*-value shows the results of the pairwise comparisons in the post hoc analysis with Bonferroni adjustment in the repeated measures ANOVA. * *p* < 0.05.

**Table 3:** Repeated measures mixed ANOVA.

Index	Multivariate Effects		Sphericity		Intra-Subjects Effects Test						
	Wilks λ Age*Group	**F**	**n**	**W (p)**	**Statistic**	**Effect**	**DF**	**Sum Square**	**Mean Square**	**F**	** $\eta^{2}$ **
VCI	0.922	2.8	71	0.995 (0.838)	Sphericity assumed	Age	2	1589.0	794.5	17.7 **	0.20
						Age*group	2	268.7	134.3	2.9	0.04
PRI	0.955	1.5	67	0.838 (0.003)	Huynh-Feldt	Age	1.79	909.4	507.9	11.6 **	0.15
						Age*group	1.79	141.0	78.7	1.80	0.02
WMI	0.871	4.8 *	69	0.918 (0.060)	Sphericity assumed	Age	2	1105.8	552.9	11.1 **	0.14
						Age*group	2	627.6	313.8	6.3 **	0.08
PSI	0.959	1.4	69	0.958 (0.240)	Sphericity assumed	Age	2	2827.7	1413.8	19.4 **	0.22
						Age*group	2	196.1	98.0	1.3	0.02
IQ	0.972	0.977	71	0.902 (0.030)	Huynh-Feldt	Age	1.89	1666.0	878.8	25.5 **	0.27
						Age*group	1.89	72.2	38.0	1.1	0.01

Note. VCI: Verbal Comprehension Index, PRI: Perceptual Reasoning Index. WMI: Working Memory Index, PSI: Processing Speed Index, IQ: Full Scale Intellectual Quotient. ** *p* < 0.01, * *p* < 0.05.

## Data Availability

The data presented in this study are available on request from the corresponding author due to privacy.
